# Income-related inequalities in the association of obesity and periodontal disease: a register-based cross-sectional analysis in the Tokyo metropolitan districts

**DOI:** 10.1007/s00784-025-06638-1

**Published:** 2025-11-15

**Authors:** Natsumi Saito, Risako Mikami, Koji Mizutani, Norio Aoyama, Takanori Matsuura, Tomonari Suda, Kohei Takeda, Jun Aida, Yuichi Izumi, Takanori Iwata

**Affiliations:** 1 Department of Periodontology, Graduate School of Medical and Dental Sciences, Institute of Science Tokyo, Bunkyo-ku, Tokyo Japan; 2 Weintraub Center for Reconstructive Biotechnology, UCLA School of Dentistry, Los Angeles, CA USA; 3 Department of Advanced Biomaterials, Graduate School of Medical and Dental Sciences, Institute of Science Tokyo, Bunkyo-ku, Tokyo Japan; 4 Department of General Dentistry, Graduate School of Medical and Dental Sciences, Institute of Science Tokyo, Bunkyo-ku, Tokyo Japan; 5 Department of Education Planning, Kanagawa Dental University, Kanagawa Yokosuka-shi, Japan; 6 Department of Oral Surgery, Secomedic Hospital, Funabashi-shi, Chiba Japan; 7 Higashi Sakura Dental Clinic, Nagoya-shi, Aichi Japan; 8 Department of Dental Public Health, Graduate School of Medical and Dental Sciences, Institute of Science Tokyo, Bunkyo-ku, Tokyo Japan; 9 Oral Care and Periodontics Center, Southern Tohoku Research Institute for Neuroscience, Southern Tohoku General Hospital, Fukushima Koriyama-shi, Japan

**Keywords:** Socioeconomic status, Obesity, Periodontal disease, Health inequality, Body mass index, Income level

## Abstract

**Objectives:**

Obesity is a risk factor for periodontal disease and is associated with socioeconomic status (SES). However, it remains unclear whether SES modifies the relationship between obesity and periodontal disease. This study investigated the influence of SES on the association between obesity and periodontal disease.

**Materials and methods:**

We used multilevel Poisson regression, after adjusting for potential confounding factors including population-level SES, to analyze the body mass index (BMI) and periodontal parameters of 962 participants (mean age 58.3 years; SD: 13.8).

**Results:**

A significant association was observed between obesity and the proportion of teeth with probing pocket depth (PPD) ≥ 4 mm (ratio of means [RM]: 1.25, 95% confidence interval [CI]: 1.14, 1.37; *p* < 0.001), whereas the high-income group exhibited a significantly lower proportion of teeth with PPD ≥ 4 mm (RM: 0.84, 95% CI: 0.71, 0.998; *p* = 0.047). Interaction analysis also revealed a significant interaction between obesity and the high-income group regarding the proportion of teeth with PPD ≥ 4 mm (*p* = 0.020). The subgroup analysis demonstrated that the RM of obesity for the proportion of teeth with PPD ≥ 4 mm was higher in females than in males.

**Conclusions:**

Income-related inequalities are associated with the relationship between obesity and periodontal disease. Among obese adults, those with low- and middle-income levels may have a higher risk of periodontal disease than those with high income.

**Clinical relevance:**

Comprehensive care and oral health education may be enhanced for obese individuals in low-income populations to mitigate their elevated risk of periodontal disease.

**Supplementary Information:**

The online version contains supplementary material available at 10.1007/s00784-025-06638-1.

## Introduction

Obesity is a precursor to various non-communicable diseases, such as type 2 diabetes [1], dyslipidemia [2], hypertension [3], cardiovascular diseases [4], respiratory diseases [5], and nonalcoholic fatty liver disease [6]. Numerous studies have investigated the association between periodontal disease and obesity [7–10]. A recent umbrella review [11] encompassing the existing evidence demonstrated a positive association between these two. Few longitudinal cohort studies support these findings [12, 13]; however, there is suggestive evidence of racial differences in the association between obesity and periodontitis, with reports indicating a stronger impact of obesity on periodontitis among Europeans and Japanese than among Americans or other Asian populations [14]. However, some research suggests that there is insufficient evidence to support a causal relationship between periodontal disease and obesity [15].

In Japan, periodontal disease is the leading cause of tooth loss among adults, surpassing dental caries, and affects nearly half of the adult population (47.8%) [16]. The disease burden is especially pronounced in the elderly, aligning with Japan’s super-aging society and resulting in decreased oral function and quality of life [17]. Despite the presence of an extensive dental workforce [18], accessibility and utilization of preventive dental care remain unequal, with socioeconomic and regional factors influencing disease prevalence [19]. Obesity has also increased, showing a 15.3% rise between 1990 and 2013, although the overall prevalence remains lower than in many Western countries [20, 21]. Importantly, individuals with overweight or obesity incur higher healthcare costs and mortality risks, with studies suggesting that Asian populations face relatively higher mortality risk from obesity compared to White populations [22].

Socioeconomic status (SES) is strongly associated with health behaviors such as dietary habits, smoking, and physical activity, and is known to influence various health-related factors, including obesity [23]. Furthermore, it is associated with the prevalence and severity of oral diseases [24] and has been suggested to contribute to health inequalities. In Japan, lower SES is consistently linked to poorer periodontal health, higher obesity prevalence, and reduced preventive care-seeking behaviors [19]. Regional disparities in access to dental care exacerbate these inequalities, leading to advanced disease and poorer outcomes in underprivileged populations. Globally, the prevalence of severe periodontitis ranges from 20 to 50%, placing Japan at the upper end of this range [25]. While Japan’s obesity prevalence remains lower than that of the United States and other high-income countries, the economic impact per case is substantial [22]. Japan’s universal health insurance system helps buffer socioeconomic gaps in financial access to care, for example, by capping copayments according to income and age and providing free medical care for those receiving public assistance [26–28].

Nevertheless, socioeconomic gradients in health outcomes persist, particularly in health behaviors and the uptake of preventive services [26], a challenge shared with many Western countries and other Asian countries that also have universal health coverage [29, 30]. At the 75th World Health Assembly, 2022, the World Health Organization set a goal of reducing health inequalities in oral diseases, conditions, and health [31]. Thus, we hypothesized that not only SES and obesity independently impact oral health, but the effect of obesity on oral health may also vary according to SES.

Multiple inflammatory pathways are assumed to mediate the mechanisms linking obesity and periodontal disease. Stress is known to elevate inflammatory biomarkers [32]. Social pressure related to body image, including physical appearance associated with obesity, can contribute to stress. Additionally, low SES is considered to exacerbate everyday stress. Accordingly, individuals with obesity and low SES may have a heightened risk of worsening periodontal disease. However, no previous studies have investigated the effect of SES on the relationship between periodontal disease and obesity. Therefore, in this study, we aimed to examine the influence of SES on the association between obesity and periodontal disease in the Japanese population, using population-level SES derived from population-level income and population-level educational attainment.

## Materials and methods

### Study design and participants

In this register-based cross-sectional study, we extracted data for analysis from a comprehensive database that records information on all patients who visit the periodontal clinic of the Dental Hospital of Tokyo Medical and Dental University (TMDU), located in the center of the Tokyo Metropolitan Districts (comprising the 23 special wards of Tokyo), for their first consultation. In addition, the range of ward-level household income and educational attainment across the Metropolitan Districts is provided in Supplementary Table 1. Therefore, prior power analysis was not performed for sample size calculation. Informed consent was obtained from all participants. The study design was approved by the Dental Research Ethics Committee of TMDU (approval number: 1085). The study was conducted following the Declaration of Helsinki of 1975, as revised in 2013, and was registered with the University Hospital Medical Information Network (http://www.umin.ac.jp) (clinical trial number: UMIN000046582). The research was also conducted in accordance with the Strengthening the Reporting of Observational Studies in Epidemiology (STROBE) statement. The data of participants meeting the following inclusion criteria were used for the analysis.

### Inclusion criteria

1. Individuals who visited the periodontal clinic of the Dental Hospital of TMDU for their first consultation between October 2014 and February 2016.
2. Only residents of the Tokyo Metropolitan Districts, which has a radius of approximately 15 km from the hospital, were considered for inclusion to prevent accessibility bias due to distance from the hospital.
3. Participants aged 18 years or older.

### Exclusion criteria

1. Participants with missing values for the dependent variable or any of the exposure/covariate variables.
2. Participants requiring a proxy for medical interviews or informed consent.

We limited the study population to residents living within approximately 15 km of the hospital to minimize accessibility bias arising from differences in travel time and cost, which may influence healthcare utilization and periodontal status. This approach was chosen over statistical adjustment for distance because unmeasured accessibility-related factors, such as transportation availability and time constraints, could not be fully accounted for.

Between October 2014 and February 2016, a total of 3,310 individuals attended their first consultation at the periodontal clinic of the Dental Hospital of TMDU. Of these, 1,671 resided outside the Tokyo Metropolitan Districts and were excluded to minimize accessibility bias due to differences in travel distance. Among the remaining 1,639 individuals, 677 were excluded because of missing data for the dependent variable or for any of the exposure/covariate variables. Consequently, 962 participants (302 men and 660 women; mean age, 58.3 years; SD, 13.8) were included in the final analysis.

### Clinical examination

Information on participants’ medical and social backgrounds was collected through medical interviews and health insurance records. Several experienced periodontists from the Department of Periodontology conducted the dental examinations, which involved recording the number of remaining teeth, probing pocket depth (PPD), and bleeding on probing (BOP). A manual probe (15 UNC Color-Coded Probe, Hu-Friedy, USA) was used for examination. The PPD and BOP were evaluated at six points on each tooth, recording the deepest PPD and the presence or absence of BOP for each tooth.

Medical histories (e.g., diabetes, smoking habits, and prior periodontal therapy) were collected through medical interviews. BMI categories were defined according to the Japan Society for the Study of Obesity (JASSO): underweight (< 18.5 kg/m²), normal weight (18.5–24.9 kg/m²), and obese (≥ 25 kg/m²). This classification differs from the WHO criteria, which define obesity as BMI ≥ 30 kg/m² and overweight as BMI ≥ 25 kg/m². Because the average BMI in the Japanese population is relatively low and individuals with a BMI ≥ 30 are uncommon, participants with a BMI ≥ 25 were treated as obese without further subdivision. Smoking status was classified as follows: never smoked, former smoker, or current smoker. Individuals who reported scaling and root planing, or those who were referred by their primary dentists, were identified as having a history of periodontal therapy.

Information on place of residence and type of health insurance was obtained from participants’ registration data in their hospital records. Population-level income and population-level educational attainment were used to determine SES.

Based on a previous study [33], we used population-level income, defined as the average household income of each participant’s residential ward, since individual-level data were not available. Thus, SES in this study was assessed at the population level using area-based indicators. We calculated the average household income of the residents of the 23 wards of Tokyo as (taxable income)/(number of taxpayers) using data obtained from a survey conducted in 2019 by the Japanese Ministry of Internal Affairs and Communications (https://www.soumu.go.jp/main_sosiki/jichi_zeisei/czaisei/czaisei_seido/ichiran09_19.html). Population-level income was divided into tertiles (low, middle, and high) and expressed in units of 1,000 yen; 1,000 yen was converted to 6.6 US dollars according to the exchange rate as of January 2024.

Similarly, population-level educational attainment was divided into tertiles (low, middle, and high) based on the proportion of residents with ≥ 16 years of formal education (university or higher) in each ward. These data were derived from the 2010 survey conducted by the Japanese Ministry of Internal Affairs and Communications (e-stat, https://www.e-stat.go.jp/, in Japanese), which was the most recent ward-level dataset available at the time of analysis; the next census with comparable data was conducted in 2020.

Population-level income and educational attainment were strongly correlated across the 23 wards (Spearman’s ρ = 0.89, *p* < 0.001). Therefore, to capture a more comprehensive measure of socioeconomic context while accounting for this correlation, we constructed a Composite area-SES index (CASI) based on both population-level income and educational attainment. For each ward, we ranked both the average household income and the proportion of residents with ≥ 16 years of education from 1 (lowest) to 23 (highest). An SES-score was then calculated as the sum of the two ranks (range 2–46). Finally, the SES-score was divided into tertiles to define the three CASI categories: low, middle, and high.

### Statistical analysis

Descriptive statistics were presented, indicating the mean (standard deviation [SD]) for continuous variables and the number (%) for categorical variables. Comparison among the three BMI-based groups was performed using a one-way analysis of variance or Fisher’s exact test.

The dependent variable was the proportion of teeth with PPD ≥ 4 mm relative to the total number of remaining teeth. The calculation of the proportions of teeth with PPD ≥ 4 mm relative to the total remaining teeth was conducted as follows: (number of teeth with PPD ≥ 4 mm)/(total number of teeth) × 100 (%). Multilevel Poisson regression analyses were employed to determine the ratio of means (RM) [34] for the proportion of teeth with PPD ≥ 4 mm, given a skewed distribution of the outcome [34]. The data were structured into multilevel models to account for participants nested in their ward of residence. In the multivariate analysis, the exposure variables were BMI, income, education level, and CASI. Four multilevel Poisson regression models were constructed. All models included the covariates age, sex, diabetes, smoking status, history of periodontal therapy, and number of remaining teeth as potential confounders. In Model 1, the exposures were BMI and income, while Model 2 included BMI and education level. Model 3 incorporated BMI and CASI, and Model 4 was based on Model 1 with the addition of an interaction term between BMI and income. The goodness of fit for the models was evaluated using the Akaike information criterion (AIC) and the Bayesian information criterion (BIC).

Statistical analyses were conducted using Stata software (version 17.0; Stata Corp LP, College Station, Texas, USA), with statistical significance set at *p* < 0.05.

## Results

Table 1 presents the demographic data, revealing that the mean age of the 962 participants (302 males and 660 females) was 58.3 years (SD: 13.8). The mean BMI for the underweight, normal weight, and obese groups were 17.3 (SD: 1.1), 21.7 (SD: 1.8), and 27.7 (SD: 2.5) kg/m^2^, respectively. The mean population-level income for the high-, middle-, and low-income groups were 8061.6 (SD: 2331.4), 4747.2 (SD: 521.9), and 3665.6 (SD: 128.3) thousand yen, respectively. The mean number of remaining teeth was 24.6 (SD: 4.8), with mean proportions of teeth having PPD ≥ 4 mm at 30.8% (SD: 28.3%). The mean proportion of teeth with BOP was 51.3% (SD: 31.3%). Significant differences were observed among the three BMI-based groups in terms of sex, BMI, diabetes, teeth with PPD ≥ 4 mm, and BOP-positive teeth.

**Table 1:** Characteristics of participants (*N* = 962)

	ALL	BMI			*p*-value*
		Underweight (*n* = 127)	Normal (*n* = 651)	Obesity (*n* = 184)	
	*N* (%) or mean (SD)	*N* (%) or mean (SD)	*N* (%) or mean (SD)	*N* (%) or mean (SD)	
Age (yrs)	58.3 (13.8)	58.1 (13.8)	59.0 (14.7)	58.3 (14.0)	0.81
Sex					
Female	660 (68.6%)	115 (90.6%)	444 (68.2%)	101 (54.9%)	< 0.001
Male	302 (31.4%)	12 (9.5%)	207 (31.8%)	83 (45.1%)	
BMI (kg/m^2^)	22.3 (3.6)	17.3 (1.1)	21.7 (1.8)	27.7 (2.5)	< 0.001
Diabetes					
Yes	73 (7.6%)	3 (2.4%)	43 (6.6%)	27 (14.8%)	< 0.001
Income					
Low	307 (31.9%)	33 (26.0%)	218 (33.5%)	56 (30.4%)	0.55
Middle	476 (49.5%)	69 (54.3%)	313 (48.1%)	94 (51.1%)	
High	179 (18.6%)	25 (19.7%)	120 (18.4%)	34 (18.5%)	
Education level					
Low	333 (34.6%)	36 (28.4%)	238 (36.6%)	59 (32.1%)	0.11
Middle	320 (33.3%)	43 (33.9%)	204 (31.3%)	73 (39.7%)	
High	309 (32.1%)	48 (37.8%)	209 (32.1%)	52 (28.3%)	
CASI					
Low	333 (34.6%)	36 (28.3%)	238 (36.6%)	59 (32.1%)	0.24
Middle	394 (41.0%)	54 (42.5%)	256 (39.3%)	59 (32.1%)	
High	235 (24.4%)	37 (29.1%)	157 (24.1%)	41 (22.3%)	
Smoking					
Never-smoker	598 (62.3%)	83 (65.4%)	416 (63.9%)	99 (53.8%)	0.11
Former smoker	265 (27.6%)	30 (23.6%)	171 (26.3%)	64 (34.8%)	
Current smoker	99 (10.3%)	14 (11.0%)	64 (9.8%)	21 (11.4%)	
**Dental health status**					
Number of teeth	24.6 (4.8)	24.6 (4.5)	24.7 (4.8)	24.5 (5.1)	0.86
Teeth with PPD ≥ 4 mm (%)	30.8 (28.3)	24.4 (26.1)	29.8 (27.5)	39.0 (30.5)	< 0.001
Teeth with PPD ≥ 6 mm (%)	12.0 (17.8)	8.1 (13.5)	11.5 (17.2)	16.2 (21.4)	< 0.001
Teeth with BOP+ (%)	51.3 (31.3)	46.9 (30.1)	50.1 (31.3)	56.8 (31.6)	0.017
History of periodontal therapy					
Yes	361 (37.5%)	43 (33.9%)	258 (39.6%)	60 (32.6%)	0.15
* One-way analysis of variance or Fisher’s exact test					

*SD* standard deviation, *BMI* body mass index, *CASI* Composite area-SES index, *PPD* probing pocket depth, *BOP* bleeding on probing

A multivariate Poisson regression analysis showed that BMI and income were associated with the proportion of teeth with PPD ≥ 4 mm (Table 2). Participants with obesity exhibited a significantly higher ratio of teeth with PPD ≥ 4 mm compared to those with normal BMI (RM: 1.25, 95% confidence interval [CI]: 1.14, 1.37; *p* < 0.001 in multivariate model 1 to 3). The high-income group had a significantly lower ratio of teeth with PPD ≥ 4 mm (RM: 0.84, 95% CI: 0.71, 0.998; *p* = 0.047, multivariate model 1) compared to the low-income group. When population-level educational attainment (multivariate model 2) or CASI (multivariate model 3) was used as the exposure instead of income, the high groups also showed lower RMs for the proportion of teeth with PPD ≥ 4 mm compared with the low groups (Model 2: RM: 0.89, 95% CI: 0.77–1.03; *p* = 0.11; Model 3: RM: 0.87, 95% CI: 0.74–1.02; *p* = 0.087). However, these differences did not reach statistical significance.

**Table 2.** Multilevel poisson regression analysis of the factors influencing the proportion of teeth with PPD ≥ 4 mm

	N	% of teeth with PPD ≥ 4 mm mean (SD)	Crude model			Multivariate model*													
						Model 1			Model 2				Model 3				Model 4		
			RM	95% CI	*p*-value	RM	95% CI	*p*-value	RM	95% CI	*p*-value		RM		95% CI	*p*-value	RM	95% CI	*p*-value
BMI																			
< 18.5: underweight	127	24.4 (26.1)	0.83	0.69, 1.00	0.048	0.85	0.70, 1.03	0.10	0.85	0.70, 1.03	0.10		0.85		0.70, 1.03	0.10	1.01	0.71, 1.44	0.95
18.5–24.9: normal	651	29.8 (27.5)	Reference			Reference			Reference				Reference				Reference		
≥ 25: obesity	184	40.0 (30.5)	1.30	1.17, 1.44	< 0.001	1.25	1.14, 1.37	< 0.001	1.25	1.14, 1.37	< 0.001		1.25		1.14, 1.37	< 0.001	1.34	1.145, 1.56	< 0.001
Income																			
Low	307	32.8 (29.5)	Reference			Reference											Reference		
Middle	476	30.7 (28.2)	0.93	0.78, 1.11	0.40	0.95	0.81, 1.13	0.58									0.98	0.83, 1.17	0.85
High	179	27.7 (25.9)	0.82	0.70, 0.97	0.021	0.84	0.71, 0.998	0.047									0.94	0.75, 1.18	0.58
Education Level																			
Low	333	32.4 (29.3)	Reference						Reference										
Middle	320	31.9 (28.9)	0.98	0.79, 1.22	0.86				1.00	0.81, 1.24	0.98								
High	309	28.0 (26.3)	0.86	0.75, 0.99	0.037				0.89	0.77, 1.03	0.11								
CASI																			
Low	333	32.4 (29.3)	Reference										Reference						
Middle	394	31.5 (28.9)	0.96	0.78, 1.17	0.65								1.00		0.83, 1.21	0.98			
High	235	27.5 (25.4)	0.83	0.72, 0.97	0.015								0.87		0.74, 1.02	0.087			
BMI x Income																			
Underweight x Middle Income																	0.77	0.50, 1.18	0.22
Underweight x High Income																	0.77	0.50, 1.20	0.26
Obesity x Middle Income																	0.99	0.82, 1.19	0.89
Obesity x High Income																	0.70	0.52, 0.94	0.020

*: Adjusted for age, sex, diabetes, smoking, history of periodontal therapy, and number of teeth

*RM* ratio of means,* CI* confidence interval, *BMI* Body Mass Index, *PPD* probing pocket depth, *CASI* Composite area-SES index

Crude model: no covariates. Model 1: BMI and income, adjusted for the above covariates. Model 2: BMI and education level, adjusted for the above covariates. Model 3: BMI and CASI, adjusted for the above covariates.

Model 4: BMI, income, and BMI × income interaction, adjusted for the above covariates.

Additionally, an examination of the interaction between BMI and income with the proportion of teeth having PPD ≥ 4 mm revealed significant interactions in the obesity and high-income group towards the ratio of teeth with PPD ≥ 4 mm (Table 2, multivariate model 4, *p* = 0.020).

**Fig. 1:**
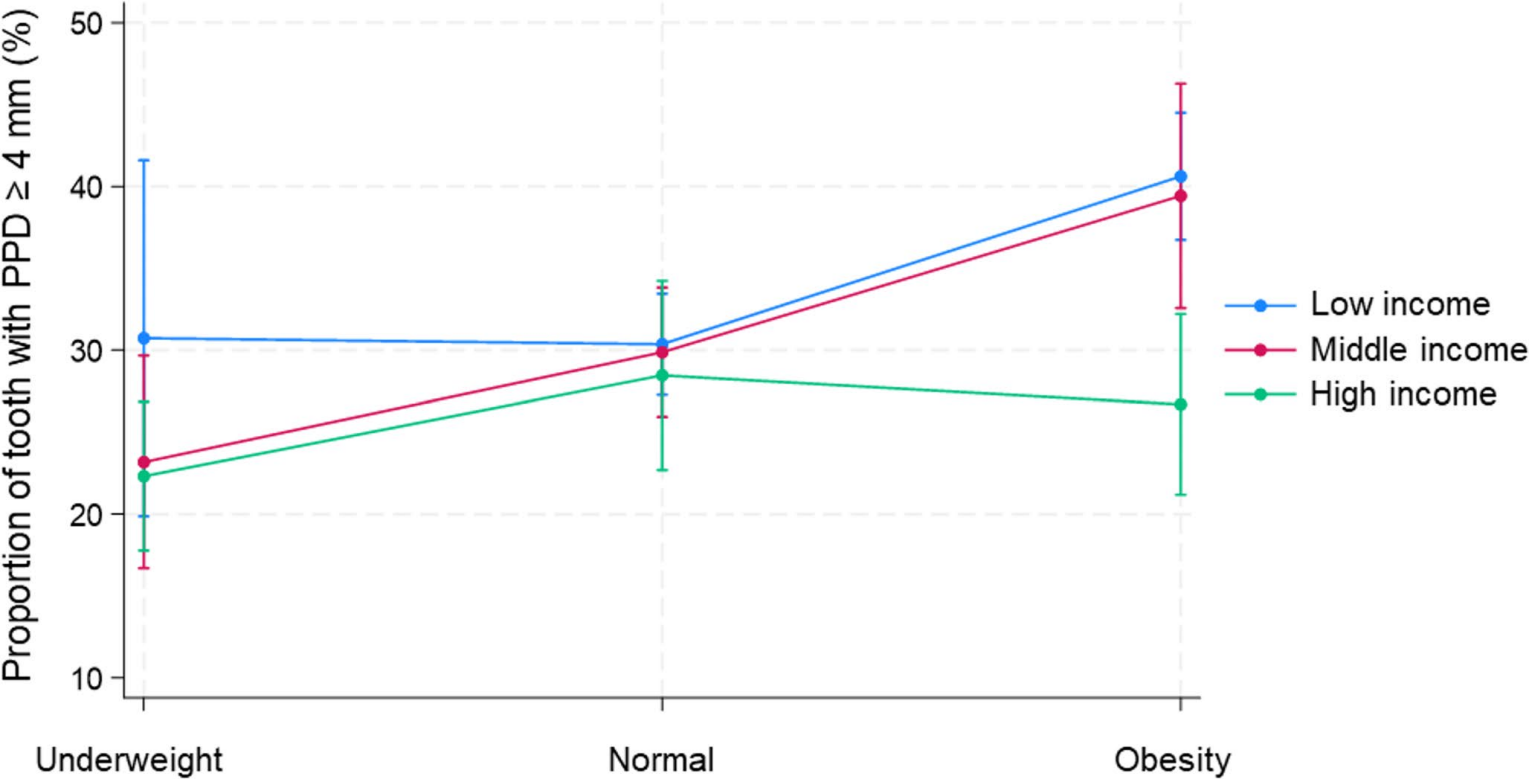
Predictive margin plot modeled based on the multivariate analyses to assess the interaction between income and body mass index (BMI) on the proportion of teeth with probing pocket depth (PPD) ≥ 4 mm

Proportions of teeth with PPD ≥ 4 mm were estimated from the models with adjustments for covariates (Fig. 1). Among participants in the high-income group, the prevalence of teeth with PPD ≥ 4 mm did not differ based on BMI. However, in the low- and middle-income group, it was observed that participants with obesity had a significantly higher proportion of teeth with PPD ≥ 4 mm. The multivariate model 4, which included the interaction terms, demonstrated the best fit according to AIC and BIC (Supplementary Table 2).

A subgroup analysis was conducted to explore sex differences in the association between obesity and periodontal disease. Although the interaction between obesity and sex towards the proportion of teeth with PPD ≥ 4 mm was not significant, the RM of obesity for the proportion of teeth with PPD ≥ 4 mm showed a trend towards a higher RM of 1.30 (95% CI: 1.10, 1.53) in females compared to 1.12 (95% CI: 0.99, 1.27) in males (Table 3).

**Table 3.** Adjusted associations between BMI and the proportion of teeth with PPD ≥ 4 mm by sex

		Male (*n* = 302)			Female (*n* = 660)		
		n (%)	RM* (95%CI)	*p*-value	n (%)	RM* (95%CI)	*p*-value
BMI							
Underweight		12 (4.0%)	0.80 (0.43, 1.47)	0.47	115 (17.4%)	0.88 (0.72, 1.07)	0.19
Normal		207 (68.5%)	1		444 (67.3%)	1	
Obesity		83 (27.5%)	1.12 (0.99, 1.27)	0.063	101 (15.3%)	1.30 (1.10, 1.53)	0.002

*Adjusted for age, diabetes, income, smoking, history of periodontal therapy, and number of teeth

*RM* ratio of means, *CI* confidence interval, *BMI* Body Mass Index

## Discussion

To our knowledge, this was the first study to demonstrate the influence of income-related inequalities on the association between obesity and periodontal disease among the Japanese population. By examining the modifying role of SES, our findings contribute to a deeper understanding of how economic factors interplay with obesity to influence oral health. Importantly, the SES indicators used in this study were derived from population-level income and educational attainment. Therefore, the observed associations should be interpreted as population-level effects, reflecting contextual effects of the socioeconomic environment of the residential area rather than individual-level status. A BMI ≥ 25 and a higher income were not only independently associated with the proportion of teeth with periodontal pockets but also showed an interaction with it. The association between obesity and periodontal disease appeared to be negligible among individuals with higher incomes, whereas it was significant for those with lower incomes. Furthermore, the association between obesity and periodontal disease tended to be stronger in females than in males after adjusting for possible confounding factors. This study has the potential to contribute to raising awareness about obesity and periodontal disease prevention among low-income individuals and improve public assistance initiatives.

Multiple mechanisms have been proposed to explain the association between obesity and periodontal disease [35]. An imbalance between pro-inflammatory adipokines produced by excess adipose tissue and anti-inflammatory adipokines leads to a systemic state of mild inflammation [36, 37]. This imbalance affects not only systemic health but also periodontal tissue destruction in obese individuals [38, 39]. Additionally, increased production of reactive oxygen species and free fatty acids by adipocytes may exacerbate inflammation and tissue destruction in periodontal tissues [40, 41]. In this study, it was observed that the proportion of teeth with PPD ≥ 4 mm increased in the group with BMI ≥ 25, suggesting that the systemic chronic inflammatory state due to obesity may impact the progression of periodontal disease.

Despite the limited consideration of body fat distribution and sex differences, BMI remains widely utilized in surveys owing to its simplicity and practicality. In Japan, a BMI ≥ 25 kg/m^2^ [42] is classified as obesity according to JASSO criteria, which differs from the WHO definition (BMI ≥ 30 kg/m²). This should be considered when comparing our results internationally. Nonetheless, several studies conducted in Japan have suggested a relationship between obesity and periodontal disease [43–46]. In both sexes, the risk of developing periodontal disease within five years is higher in the group with BMI ≥ 25 kg/m^2^ compared to the group with BMI < 22 kg/m^2^, with the risk being greater for females than males [47]. Consistent with previous findings, we also found that a BMI ≥ 25 kg/m^2^ was associated with a higher risk of periodontal disease among females compared to males. Several studies have proposed possible underlying mechanisms. A study reported that in females, a high body adipose index combined with a low skeletal muscle index was significantly associated with an elevated odds ratio of periodontal disease by utilizing Dual-energy X-ray Absorptiometry, which allows for a more precise measurement of obesity than BMI [48]. However, it has been observed that females face higher social pressures [49] regarding their appearance, including body shape, than males. Consequently, females may experience greater stress related to being overweight, a condition that may be implicated in the progression of periodontitis. Evidence suggests that stress significantly impacts periodontitis [50]. However, the mechanisms underlying sex-related differences in the association between obesity and periodontal disease remain unclear.

Interestingly, we found a statistically significant difference in the adverse effects of obesity on the proportion of teeth with periodontal pockets depending on income level. This study is the first to show a significant interaction between obesity, income, and the proportion of teeth with periodontal pockets. The relationship between SES and health is commonly mediated by lifestyle factors such as alcohol consumption, lack of exercise, and stress [51, 52]. These factors, such as those not considered in this study, may be reflected in the results. Another hypothesis stated that high-income populations engage in meticulous oral hygiene and regular dental care. Individuals with a high SES generally have high health literacy [53, 54]; moreover, high-income participants may be better able to pay for the costs of oral hygiene and dental care. These positive oral health behaviors may compensate for the adverse effects of obesity in those in the high-income group. Furthermore, a prior study noted that physical appearance related to obesity may impact employment, potentially affecting income [55]. Although the temporal relationship between income and obesity is unclear in this study, individuals with both low income and obesity may experience compounded stress from both factors, which could contribute to the progression of periodontal disease.

Another interesting point is that our findings suggest that income may play a greater role than education level in SES-related differences. Previous studies in Japan, however, have reported mixed results. Abbas et al. [56] demonstrated that high income was more strongly associated with dental implant usage than high education among elderly individuals, and Tashiro et al. [57] found that community-level income inequality was linked to fewer remaining teeth in older adults, although their study was limited to Niigata City. In contrast, a nationwide analysis by Taira et al. [19] suggested that educational attainment exerted a stronger influence than income on regional disparities in dental care utilization. These inconsistencies suggest that the relative importance of income and education may vary based on the outcome, geographic scope, and study population.

International studies from the United States, Europe, and Korea have likewise shown that the odds of obesity and periodontitis are higher among socioeconomically disadvantaged groups, despite differences in cultural norms, social structures, and health insurance systems [58–60]. Our findings extend this body of evidence by providing, to our knowledge, the first evidence in a Japanese population showing that area-level socioeconomic status modifies the association between obesity and periodontal disease, with the adverse effect of obesity pronounced in the low- and middle-income groups but attenuated in the high-income group.

This study had several limitations. First, there are limitations related to data collection. Due to the unavailability of individual income and educational attainment data, population-level income and educational attainment were used instead, derived from residential areas. This may not accurately reflect the participants’ actual SES. While discrepancies exist between individual and area-level SES, the bias introduced by this method is considered nonsystematic rather than systematic. Therefore, the results of this study may have a wider CI, and the observed significant results may still be robust. The use of area-level SES implies the presence of contextual effects within residential environments. However, demonstrating such effects requires analysis that incorporates both area-level and individual-level SES. Future studies should employ both indicators to more robustly assess contextual effects. Another limitation of this method is that the results may be associated with both income and education level disparities, as well as other factors, such as the local environment. Further research using individual SES data is, thus, required to validate the results of this study. The use of ward-level educational attainment data from 2010 may also be a limitation, as it preceded our 2014–2016 study period by several years. However, educational attainment in Japan tends to change slowly, and ward-level rankings remain relatively stable across census periods. Therefore, we consider that the use of 2010 data is unlikely to have substantially affected our findings. Another limitation concerns the representativeness of the study population. We included only residents living within approximately 15 km of the hospital to minimize accessibility bias from differences in travel time and cost, which could influence healthcare utilization and periodontal status. This restriction resulted in the exclusion of 1,671 individuals, representing 50.5% of all first-time visitors during the study period. These excluded individuals may differ systematically from included participants in terms of socioeconomic characteristics, health behaviors, and periodontal status, potentially limiting the generalizability of our findings to populations in more distant or rural areas. Therefore, the magnitude of socioeconomic inequalities observed here may be conservative. Moreover, detailed data on smoking habits, such as the duration of smoking and the number of cigarettes consumed per day, are missing. While smoking status was categorized as “current smoker,” “former smoker,” and “never smoked,” we acknowledge that this classification does not fully account for the intensity of smoking, which is a key periodontal risk factor. Furthermore, height and weight were self-reported by participants. Although self-reported body weight tends to be underestimated, such misclassification is considered non-differential across income levels and would bias the association toward the null. Given that significant associations were still observed, the influence of this bias is likely limited. Future studies should thus incorporate more comprehensive smoking data for a more detailed analysis. Another limitation regarding data collection is the inclusion of PPD ≥ 4 mm that may not be associated with active periodontitis, such as inactive pockets following successful periodontal therapy or pockets caused by non-periodontal factors, including root fractures. Given that the mechanism by which obesity influences periodontal disease is linked to inflammation, it is plausible that the interaction between obesity and SES would be more pronounced in active periodontal pockets. If an analysis limited to active periodontal pockets could be performed, stronger associations might be observed. Therefore, the inclusion of inactive pockets in this study likely introduced a bias that underestimated the true strength of the relationship. Nevertheless, the significant interaction between obesity and SES observed in this study suggests that the findings could be robust. Future studies should, thus, refine the classification of periodontal disease activity to account for these variations and further validate the results. Second, we adjusted for diabetes as it is a known confounder affecting both obesity and periodontal disease in this study. However, other systemic diseases that could potentially influence both conditions were not specifically adjusted for. Third, because this was a cross-sectional study, causality could not be determined. Cohort and life course studies should thus investigate the impact of SES more thoroughly in the future. Fourth, a preliminary power analysis was not conducted as this was an observational study using a database. The possibility of underestimation owing to the small sample size is present because of the lack of power analysis; however, the sample size of this study was considered sufficient to demonstrate significant differences. As additional examinations beyond those routinely performed in clinics were not necessary for this study, obtaining a large sample size without ethical concerns improved the generalizability of the study results. Fifth, because the data were collected during routine clinical practice, the pre-study calibration of the examiners was not performed in this study. Although all examiners were experienced periodontists, who had completed the same postgraduate education and clinical training, the lack of calibration could lead to non-differential misclassification, potentially leading to bias, widening the confidence intervals. Despite this potential bias, the detection of statistically significant differences suggests that the results of the analysis could be robust. Lastly, a notable limitation of this study is the absence of Clinical Attachment Level (CAL) measurements, which are crucial for accurately assessing periodontitis. The lack of CAL data raises the possibility of overestimating the severity of periodontal disease. Future studies should incorporate CAL measurements to ensure a more precise evaluation of periodontitis.

## Conclusions

A significant association between obesity and the proportion of teeth with periodontal pockets was demonstrated in this study. The high-income group had a significantly lower proportion of teeth with PPD ≥ 4 mm compared to the low- and middle-income groups. Furthermore, among adults with obesity, those with low- and middle-income levels may face a higher risk of periodontal disease than those with high income.

## Supplementary Information

Below is the link to the electronic supplementary material.

Supplementary Material 1 (DOCX 17.2 KB)

Supplementary Material 2 (DOCX 18.6 KB)

## Data Availability

The datasets generated and/or analyzed in the current study are available from the corresponding author upon reasonable request.
