# Supplementary material for: Income-related inequalities in the association of obesity and periodontal disease: a register-based cross-sectional analysis in the Tokyo metropolitan districts
Source: Clin Oral Investig. 2025 Nov 15;29(12):570. doi: 10.1007/s00784-025-06638-1 (PMC12619761; doi:10.1007/s00784-025-06638-1)
Supplement: Supplementary file 1 — Supplementary Material 1 (DOCX 17.2 KB) [file 784_2025_6638_MOESM1_ESM.docx]

| **Supplementary Table 2.** Goodness of fit of the models in Table 2 | | | |
| --- | --- | --- | --- |
|  |  | AIC | BIC |
| Crude model | |  |  |
|  | BMI | 28543.7 | 28563.2 |
|  | Income | 29064.0 | 29078.6 |
|  | Education level | 29065.4 | 29084.9 |
|  | CASI | 29065.3 | 29084.8 |
| Multivariate model | |  |  |
|  | Model 1 | 26646.9 | 26724.8 |
|  | Model 2 | 26647.8 | 26725.7 |
|  | Model 3 | 26647.3 | 26725.2 |
|  | Model 4 | 26533.4 | 26630.8 |
| AIC, Akaike Information Criterion; BIC, Bayesian Information Criterion; BMI: Body Mass Index, CASI, Composite area-SES index. | | | |
