# Supplementary material for: Income-related inequalities in the association of obesity and periodontal disease: a register-based cross-sectional analysis in the Tokyo metropolitan districts
Source: Clin Oral Investig. 2025 Nov 15;29(12):570. doi: 10.1007/s00784-025-06638-1 (PMC12619761; doi:10.1007/s00784-025-06638-1)
Supplement: Supplementary file 2 — Supplementary Material 2 (DOCX 18.6 KB) [file 784_2025_6638_MOESM2_ESM.docx]

| **Supplementary Table 1. Socioeconomic characteristics of residents in the 23 special wards of Tokyo** | | | | |
| --- | --- | --- | --- | --- |
| **Ward** | **n** | **Average annual household income in thousand yen** | **% with university degree or higher** | **Proportion of teeth with PPD ≥ 4mm mean (SD)** |
| Adachi | 54 | 3470 | 13.75 | 36.3 (29.5) |
| Arakawa | 27 | 3740 | 19.62 | 33.5 (34.9) |
| Bunkyo | 68 | 6230 | 35.59 | 31.0 (25.8) |
| Chiyoda | 11 | 10820 | 36.71 | 33.7 (29.8) |
| Chuo | 20 | 6900 | 35.82 | 20.3 (28.5) |
| Edogawa | 69 | 3670 | 17.48 | 30.0 (27.5) |
| Itabashi | 33 | 3730 | 21.1 | 31.8 (29.4) |
| Katsushika | 44 | 3540 | 16.41 | 33.3 (28.3) |
| Kita | 39 | 3750 | 21.62 | 24.6 (25.7) |
| Koto | 56 | 4490 | 25.7 | 46.0 (30.0) |
| Meguro | 21 | 6370 | 30.07 | 29.4 (28.9) |
| Minato | 33 | 12170 | 31.29 | 23.7 (24.7) |
| Nakano | 31 | 4280 | 27.65 | 26.8 (23.6) |
| Nerima | 80 | 4260 | 25.88 | 30.8 (28.3) |
| Ota | 27 | 4360 | 24.72 | 28.7 (26.4) |
| Setagaya | 74 | 5690 | 31.08 | 33.9 (31.5) |
| Shibuya | 26 | 8730 | 29.27 | 26.4 (23.8) |
| Shinagawa | 21 | 4970 | 26.64 | 18.7 (22.2) |
| Shinjuku | 47 | 5410 | 27.18 | 25.8 (24.6) |
| Suginami | 56 | 4720 | 36.24 | 36.8 (29.4) |
| Sumida | 41 | 3870 | 19.72 | 34.8 (29.5) |
| Taito | 26 | 4330 | 21.41 | 27.7 (27.5) |
| Toshima | 58 | 4490 | 22.72 | 29.0 (28.5) |
